# Objective perimetry and diabetic retinopathy progression: a 10-year follow-up study

**DOI:** 10.3389/fendo.2025.1755262

**Published:** 2026-01-12

**Authors:** Betelhem T. Yibekal, Bhim B. Rai, Joshua P. van Kleef, Faran Sabeti, Emilie M. F. Rohan, Christopher J. Nolan, Ted Maddess

**Affiliations:** 1 Eccles Institute for Neuroscience, The John Curtin School of Medical Research, The Australian National University, Canberra, ACT, Australia; 2 Discipline of Optometry, Faculty of Health, University of Canberra, Canberra, ACT, Australia; 3 Department of Endocrinology, The Canberra Hospital, Garran, ACT, Australia; 4 School of Medicine and Psychology, The Australian National University, Canberra, ACT, Australia

**Keywords:** diabetic retinopathy, multifocal, objective perimetry, progression, type 2 diabetes

## Abstract

**Purpose:**

We investigated 10-year retinal function changes in persons with type 2 diabetes (PWT2D) using the objectiveFIELD Analyser (OFA) to measure per-region sensitivity and response delay. We also examined which baseline measures predicted diabetic retinopathy (DR) progression.

**Methods:**

Participants underwent a comprehensive anterior and posterior segment examination at both visits. Participants were examined by four OFA test methods differing in visual field eccentricity and test duration, each producing per-region sensitivities and response delays. DR severity was graded using Early Treatment of Diabetic Retinopathy Study (ETDRS) scores. A generalized linear mixed-effects logistic regression model identified predictors of DR progression over 10 years.

**Results:**

At the 10-year follow-up, 16 PWT2D (11 men, mean age 67.3 ± 11.9 years) were reexamined and more than half (10 participants) exhibited DR progression by at least one ETDRS severity level. Average total deviations of the OFA30 test showed progressive sensitivity loss, particularly in peripheral and nasal regions, whereas response delays demonstrated radially symmetric defects for both OFA30 and OFA15. Logistic regression revealed that regional reduced sensitivity and prolonged delays were significantly associated with higher odds of DR progression, alongside clinical factors such as blood glucose, diabetes duration, biothesiometry score, and baseline DR severity. For OFA30, an initial sensitivity loss of 10 dB in the nasal field produced odds of progression of 1.74× (SE 1.50× and 2.03×, p<0.001). Initial central field delays of 10 ms increased odds by 1.12× (SE 1.08× and 1.15×, p<0.001). Linear models further confirmed that DR severity was significantly associated with changes in OFA sensitivity and response delay.

**Conclusion:**

The OFA may provide a rapid and convenient method for monitoring and predicting DR progression. By assessing changes in retinal function, OFA shows potential as a valuable tool for tracking disease progression and may offer greater sensitivity for detecting functional changes over time.

## Introduction

Some of the diagnostic techniques that may be used to diagnose classical vascular diabetic retinopathy (DR) include direct and indirect ophthalmoscopy, fundus photography, optic coherence tomography (OCT), and optic coherence tomography-angiography (1). It is becoming clear that structural and functional changes can precede classical retinopathy in diabetic retinal disease (DRD) (2, 3). When measured in terms of diagnostic power, perhaps the best method is a new form of objective perimetry, the objectiveFIELD Analyser^®^ (OFA^®^) (2). A novel feature of the OFA is that it reports both: sensitivity changes, and changes in response delay at every visual field region (4–7). OFA also reports on abnormally low and high sensitivity, and faster and slower than normal delays. The transient yellow OFA stimuli ensure it is accessing cortically driven responses (8–10). Studies using standard automated perimetry (SAP) have reported higher than normal mean defects in early-stage DRD (11). Higher than normal per-region sensitivities (hypersensitivity) have also been reported in early glaucoma for flickered SAP stimuli (12). In neovascular age-related macular degeneration (AMD), OFA peripheral-macular hypersensitivity predicts good responses to initial antivascular endothelial growth factor (VEGF) treatment (13) and even the need for treatment (14). AMD severities are well correlated with variability in two-dimensional macular pigment optical density (MPOD). The correlation between MPOD and OFA sensitivities and delays differed with severity stage, indicating that they convey different information about disease progression. Two cross-sectional OFA studies of early DRD suggested that hypersensitivity and short delays were associated with fewer years of disease, whereas later-stage disease was associated with lower sensitivity and long delays (5, 7). Overall, the relative independence of OFA sensitivity and delay measurements may suggest they are measuring different aspects of ocular disease.

Support for this idea comes from several OFA longitudinal studies, where progression of sensitives and delays can be quite independent in time and space. This has been reported in glaucoma over 5 years (15) and multiple sclerosis over 10 years (16). In a 15-month study of exudative AMD patients on *prn* anti-VEGF management, the need for treatment was differentially predicted by changes in peripheral macular delays and sensitivities (14). A 27-month study of diabetic macular oedema showed that changes in per-region delays were the best predictor of progression or recovery of diabetic macular oedema, whereas matrix perimetry provided no information on progression. Here, we reexamine a group of persons with DRD who were tested with two different OFA tests 10 years earlier. That study, reported in this journal, showed very favourable results compared with Matrix or Short Wavelength Automated perimetry (SWAP) (7).

A recent feature of OFA is the introduction of three 5th-generation, high-efficiency tests that assess both eyes in under 90 s (17, 18). We examine two such tests: a wide-field and a macular OFA version. These closely match the visual field extents of the two higher spatial resolution 4th-generation tests examined here 10 years apart. We will compare the tests. Our main objective is to use 10-year retinal function changes in persons with type 2 diabetes (PWT2D) to examine the prognostic power for DR of OFA per-region sensitivity and response delays.

## Methods

### Subjects

In this longitudinal study, 16 PWT2D who were initially tested in our clinic were reexamined after 10 years. Participants who had ophthalmological or neurological diseases that would potentially affect visual acuity, colour vision, visual fields, or pupillary function were excluded. Additional exclusion criteria included history of ocular or head trauma, uncomplicated cataract surgery within 6 months, complicated cataract surgery at any time, spherical equivalent refractive error greater than ±9.00 diopters, or cylinder worse than ±2.00 diopters. This study was approved by the Australian Capital Territory Health Human Research Ethics Committee, and the Australian National University Human Research Ethics Committee. Informed written consent was obtained from each participant. The research adhered to the tenets of the Declaration of Helsinki.

### Examinations

Participants underwent a comprehensive anterior and posterior segment examination at both visits. Best Corrected Visual Acuity was measured using a logMAR chart. Slit-lamp examination was performed to exclude media opacities, and pupillary reactions were evaluated to rule out relative afferent pupillary defect. Intraocular pressure and central corneal curvature were also measured. Visual field assessment was conducted using the Matrix perimeter (24-2 test). Subsequently, the pupils were dilated using 1% tropicamide eye drops, and OCT scans of the macula and peripapillary retinal nerve fibre layer thickness were performed. In addition, OCT angiography of the macula and optic nerve head was done. Five 45° fundus photographs were taken for DR grading. Each fundus photograph was evaluated by a senior retinal surgeon (BBR) and a senior optometrist (FS) for key features of DR such as microaneurysms, haemorrhages, drusen, intraretinal microvascular abnormalities, venous abnormalities, and neovascularisations. Based on the presence and severity of these lesions, the eye was classified according to an established grading system, the Early Treatment Diabetic Retinopathy Study (ETDRS) severity scale, and ranged between L10 to L65 (19).

Additional diabetes complication assessments included blood pressure measurement, skin autofluorescence testing, and biothesiometry. The biothesiometer is a standard device that measures vibration sensitivity on the foot to assess the degree of peripheral neuropathy. To assess the vibration perception threshold, the biothesiometer probe was placed to six locations on each foot (great toe, 1st, 3rd, and 5th metatarsal heads, instep, and heel). The voltage was gradually increased from 0 to 50 V, and the level at which the participant first perceived the vibration at each site was recorded. The biothesiometer score was calculated as the average of these threshold voltages across all six points for each foot (20). The AGE Reader is a non-invasive device that uses skin autofluorescence to estimate systemic accumulation of advanced glycation end products (AGEs) (21).

### ObjectiveFIELD analyser

Objective visual fields were measured with an FDA-cleared, ObjectiveFIELD Analyser^®^ (OFA^®^, Konan Medical USA, Irvine CA). The OFA testing was performed prior to any other ocular examinations, and participants were instructed to avoid consuming caffeinated beverages for at least 1 h before testing.

The OFA presented dichoptic multifocal stimuli simultaneously to both eyes via two liquid-crystal displays operating at 60 frames/s, with synchronised video cameras measuring direct and consensual responses from each eye concurrently (22). The responses measured are the relative constriction amplitude (converted to dB sensitivity) and time to peak (delay in ms), which is especially informative in diabetes (5, 6).

**Figure 1:**
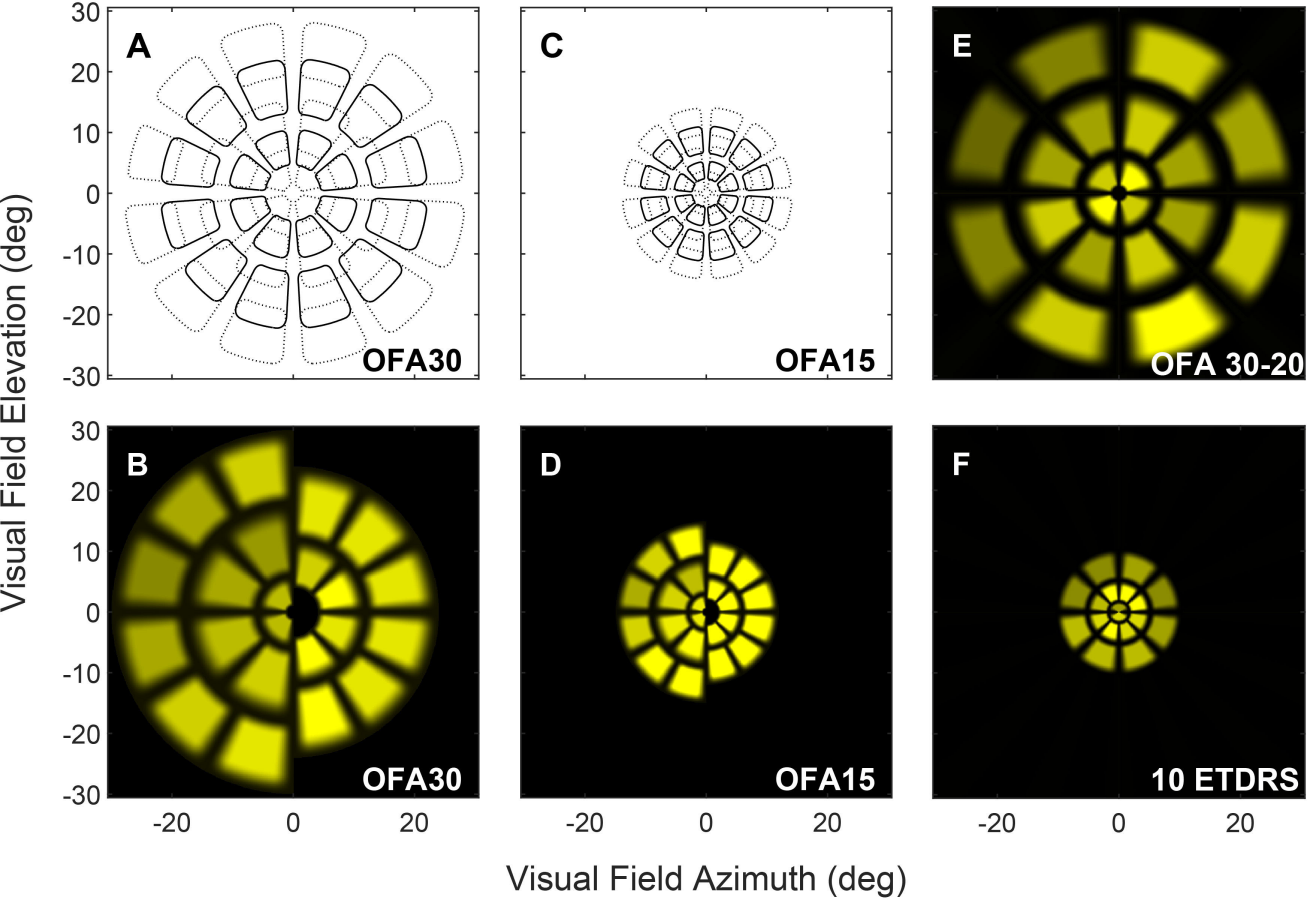
The panels show the spatial layout of the four main types of commercially available OFA stimuli, OFA30, OFA15, OFA 30-20 (a.k.a. W20), and the macular OFA 10 ETDRS (a.k.a. M18). The individual stimulus regions are presented via a multifocal stimulus method and appear transiently and randomly over time. The OFA30 and OFA15 stimulus arrays are scaled by a factor of 2. Their stimuli are potentially overlapping. **(A, C)** Boundaries of the regions. **(B, D)** Representations of the relative brightness of the left and right halves of the sets of rings of stimuli. **(E, F)** Layout of the newer W20 and M18 stimuli.

Two OFA stimulus protocols were used at both visits, OFA15 and OFA30 covering the central 30° and 60° of the visual field, respectively (5, 23). Each test takes 7–8 min, divided into nine 40-s segments with short rest breaks in between. The stimulus array was displayed in a dartboard layout consisting of 44 regions (11 per quadrant), arranged in five rings (**Figures 1A-D**). Individual stimuli presented for 33 ms with a mean interstimulus interval of 4 s at any given location, following pseudo-random temporal sequences generated using the Clustered Volleys method (24). A total of 90 stimuli were presented at each location, and the stimuli were delivered so that they never overlapped spatially. The per-region responses were calculated as the mean of the 90 presentations. The multifocal analysis was a non-linear regression method in which the response waveforms for each region were directly fitted to a log-normal model. This gave the response amplitude (which was transformed to decibel sensitivity) and the time to peak constriction (the response delay). Further details of the model are given at (25).

Additionally, two newer OFA test protocols, OFA 30-20 and OFA 10 ETDRS, were added at the second visit. The OFA 10 ETDRS is a rapid macular protocol assessing 18 regions/eye extending to ± 10° (**Figure 1F**). Its test regions and stimulus layout correspond to the EDTRS OCT subfields, allowing easy structure/function comparisons (18). The OFA 30-20 is a rapid wide-field protocol that tests 20 regions/eye across ± 30° (**Figure 1E**). Both protocols have a test duration of less than 90 s during which each region is tested 22 times (23).

The luminance of each stimulus for each protocol was designed to produce similar response amplitudes in healthy subjects, so-called luminance balancing (26). Stimulus borders were blurred allowing tolerance of ametropia up to ±3.0D. Trial lenses were used to correct participants’ distance refractive error, and stimuli were presented at optical infinity. Any vergence deficit was corrected before starting the tests. The participants fixated on a plus symbol at the centre of the viewing field. Fixation and blinks were monitored online, and data obtained during those periods were removed. Tests were repeated if data loss exceeded 15% of the total recording. The order of OFA protocols was randomized, and all protocols tested both eyes independently and concurrently.

### Data analysis

**Figure 2:**
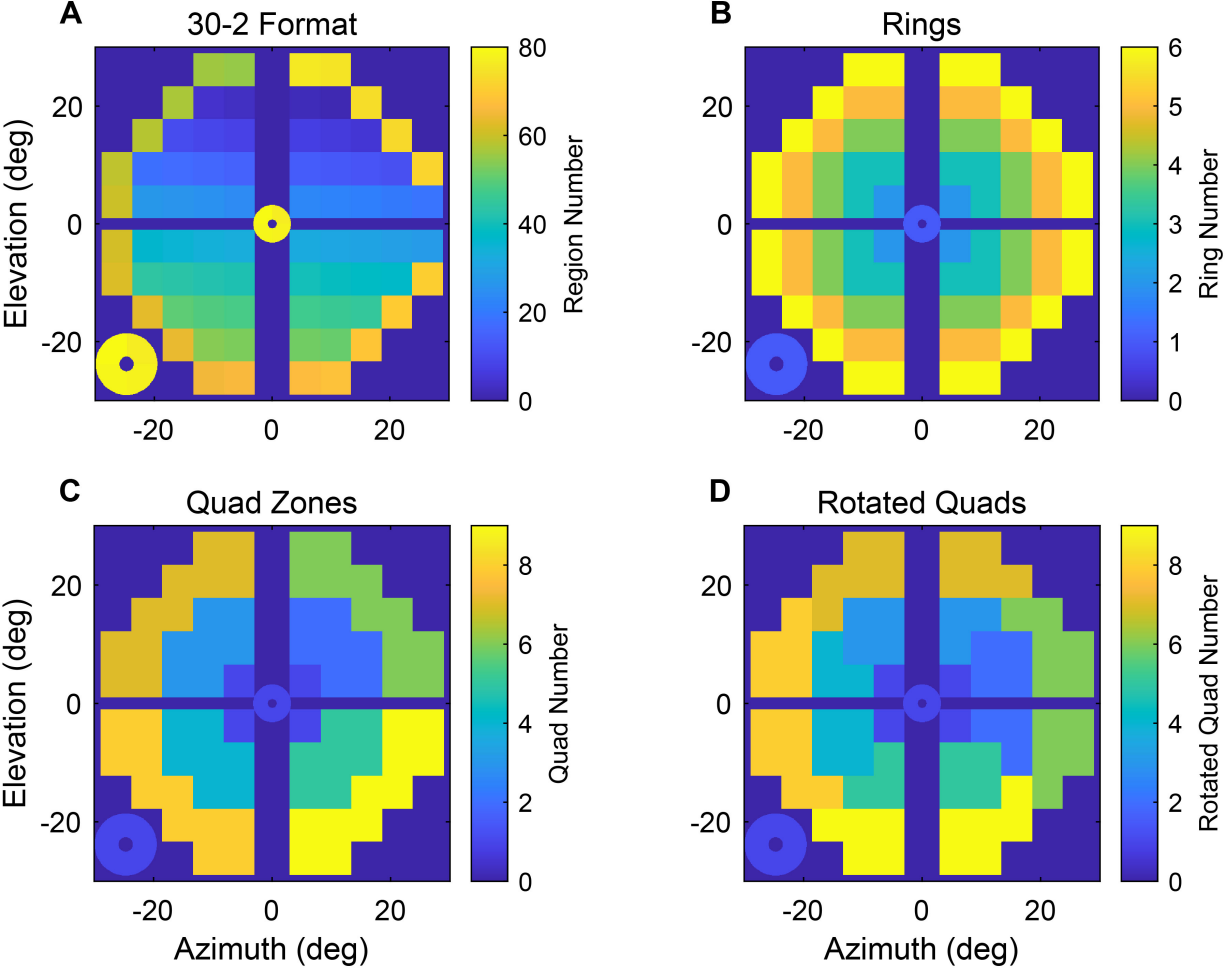
The report array and layout of collection of those regions used for statistical analysis. **(A)** The OFA30 and OFA15 data were mapped to a 30-2 pattern (half that scale for OFA15). **(B)** Layout of the six rings used as factors (categorical variables) in the mixed effects models. **(C)** The nine quadrants by ring zones (Quads). **(D)** versions of the Quad zones rotated by 45°.

Data analysis was performed using MATLAB (The MathWorks, LaGrange, GA). The response waveforms from each of the 18, 20, or 44 OFA test regions per eye were extracted from raw pupillary responses using multiple regression, with blinks removed from the pupil data before regression (27). Responses for each retinal region were fitted to a log-normal function to estimate per-region sensitivity and response delay (28). Response amplitudes were then logarithmically transformed into decibels (dB), whereas response delays (times to peak constriction) were recorded in ms. For OFA 30 and OFA 15, these were further transformed to a 30-2 pattern for reporting purposes (**Figure 2A**) (29). In addition, three alternative reporting layouts were used to analyse collections of responses (**Figures 2B-D**). Per-regional sensitivity and delay total deviations (TD) and pattern deviations (PD) from the OFA normative data were computed. Before calculating eye-wise measures, the per-region data from the right eyes were reflected to match the left-eye equivalent locations. Thus, the data from anatomically equivalent pairs of regions in the two eyes of each participant could be easily compared. The effects of OFA per-region sensitivity and delay TD and potential predictors such as age (fitted in decades relative to the mean age), sex, ETDRS severity, and visual field eccentricity on the risk (odds) of DR progression were explored using mixed-effects logistic regression models. Similar models examined the effects of OFA per-region sensitivity and delay PD. Furthermore, linear mixed-effect models were used to determine factors affecting longitudinal changes in OFA sensitivity and delay. The models contained random effects to ameliorate the possible effects of multiple comparisons. In particular, the effect of eyes within subjects was fitted. Two classes of models were fitted: standard mixed effects linear models (Matlab function *fitlme*), and models of log(odds) that used generalised mixed effects linear models (Matlab function *fitglme*) with a binomial distribution selected and a logit link function.

## Results

### Participant data

A total of 16 PWT2D (11 men) with the mean age of 67.3 ± 11.9 (mean ± SD) years participated in this study. The participants had long-standing diabetes (23.7 ± 7.2 years, range 11 to 36 years). A total of 11 participants had evidence of peripheral neuropathy (biothesiometry score >15 on both feet). The summary and participant characteristics including demographic, ocular assessments other than OFA, and diabetes complication assessments are presented in **Table 1**.

**Table 1:** Clinical and demographic characteristics of the persons with type 2 diabetes on visit 2.

Variables	Mean ± SD
Values per subject (N=16)	
Age (years)	67.3 ± 11.9
Sex (frequency (%))	
Male	11 (68.75%)
Female	5 (31.25%)
Diabetes duration (years)	23.7 ± 7.2
Hypertension (frequency (%))	
Yes	9 (56.25%)
No	7 (43.75%)
Blood pressure (mmHg)	
Systolic	136 ± 13.5
Diastolic	79.25 ± 15
Heart rate (BPM)	74.4 ± 10.3
AGE score	3.2 ± 0.6
Biothesiometer Score	30 ± 18
Values per eye (N=32)	
IOP (mmHg)	13.15 ± 2.54
Matrix mean deviation (dB)	-1.25 ± 2.88
Matrix pattern deviation (dB)	3.42 ± 1.14
BCVA (LogMAR)	0.1 ± 0.1
DR severity (frequency (%))	
ETDRS 10	16 (50%)
ETDRS 20	8 (25%)
ETDRS 35	3 (9.38%)
ETDRS 53	4 (12.50%)
ETDRS 65	1 (3.12%)

### Average OFA total deviations

**Figure 3:**
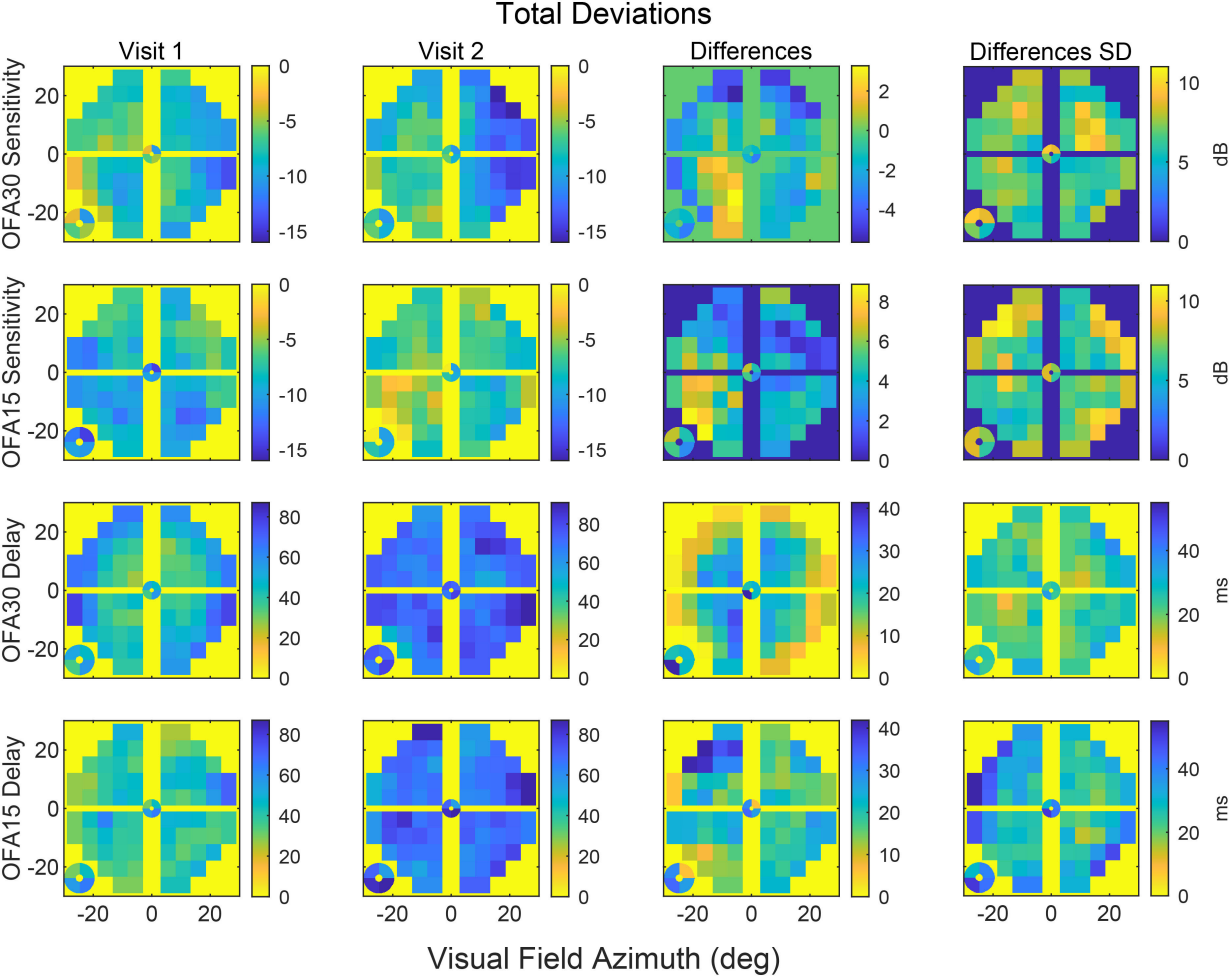
The median sensitivity and delay total deviation data for OFA30 and OFA15 for two visits, their difference, and standard deviation (SD). The medians were computed at each 30-2 field location for all eyes. From the left to right columns: visit 1 (done in 2013/14), visit 2 (2024), difference, and SD of differences. Rows top to down show OFA30 sensitivity, OFA15 sensitivity, OFA30 delay, and OFA15 delay data.

The results of OFA30 and OFA15 tests were analysed to assess the average changes in sensitivity and delays relative to the baseline test (**Figure 3**). In these plots, the yellow background indicates normal TD levels (i.e., 0), whereas cooler/darker tones represent abnormality, as shown on the calibration bars. The median TD plot from the OFA30 test showed reduced sensitivity in the lower nasal quadrant at visit 1, which appeared to progress at visit 2, where both the upper and lower quadrants showed reduced sensitivity. In the OFA15 test which corresponds to visual space covered by the inner rings of OFA30, reduced sensitivity was observed in the temporal field at visit 1, which appears to improve at visit 2. By contrast, the delay reports from both the OFA30 and OFA15 tests indicated worsening visual field defects over time. During visit 1, the more peripheral rings in the OFA30 showed longer delays compared with the inner rings. Although OFA15 exhibited less pronounced delays than OFA30 initially, after 10 years, both central and peripheral regions demonstrated prolonged delays across both stimulus protocols. The median sensitivity and delay pattern deviation plots for OFA30 and OFA15 across the two visits, along with their differences and standard deviations, are presented in **Supplementary Figure S1**.

**Figure 4:**
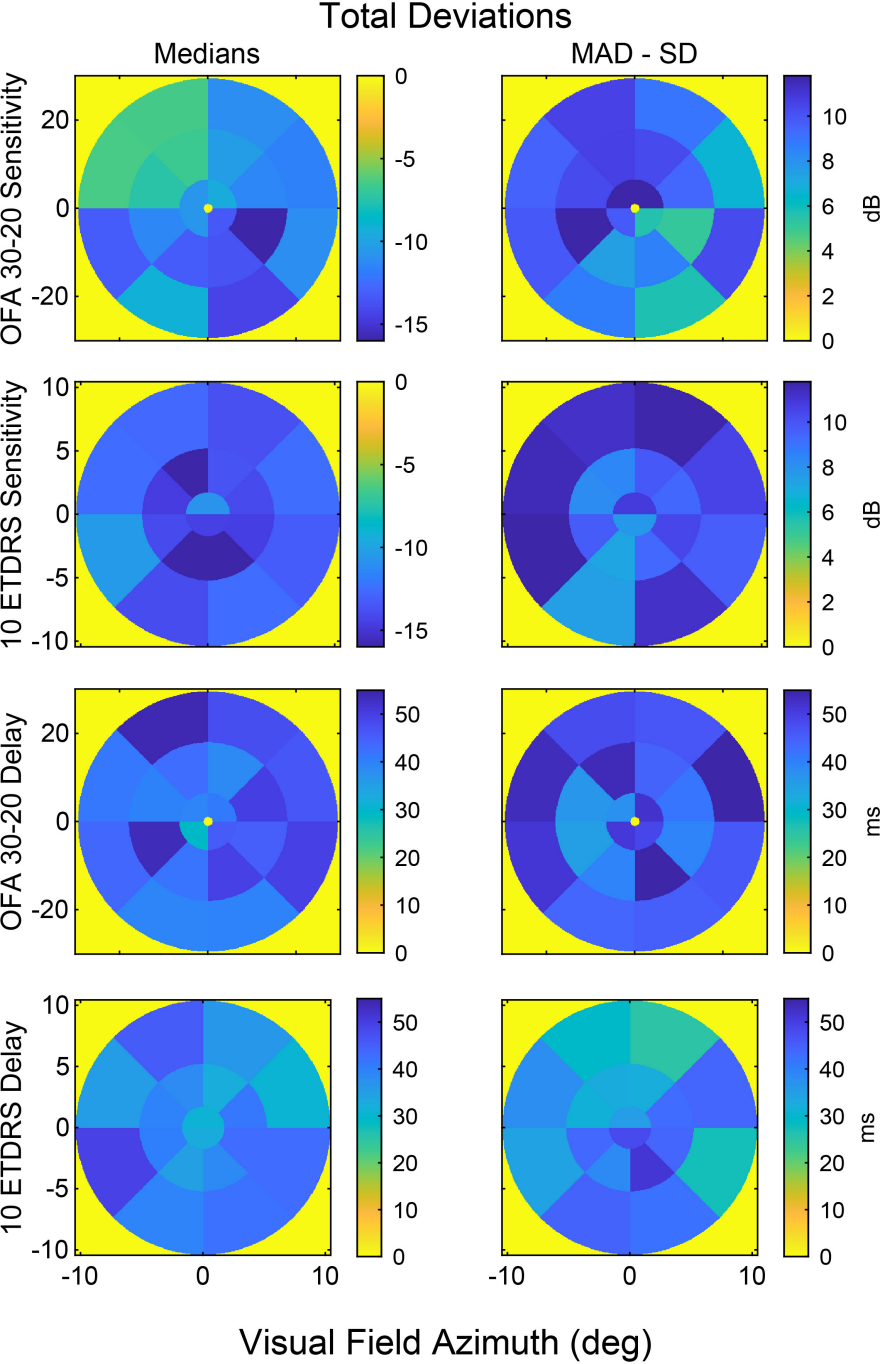
The median sensitivity and delay total deviation data for OFA30-20 and OFA10 ETDRS, and their MAD-SD (the median absolute value scaled to be the same size as the SD for normally distributed data). From left to right columns: medians and MAD-SD. Rows top to down show OFA30-20 sensitivity, OFA10 ETDRS sensitivity, OFA30-20 delay, and OFA10 ETDRS delay data.

The sensitivity and delay TD and PDs were also evaluated using the newer “High-Efficiency” OFA protocols, OFA30-20 and OFA10 ETDRS, which were only available for the second visit. **Figure 4** presents the median sensitivity and delay TD for both protocols and their median absolute value plots. In the wider field test, OFA30-20, the superior-temporal field showed relatively better sensitivity compared with other quadrants; however, the corresponding delay TD plot revealed prolonged response delays in this same region. In the more central test, OFA 10 ETDRS, reduced sensitivity was observed predominantly in the central areas, whereas the delay plot demonstrated longer response delay in the more peripheral regions, although these were less than the delays of the peripheral OFA30-20 regions (**Figure 4**). Data for the pattern deviations is presented in **Supplementary Figure S2**.

### Diabetic retinopathy progression analysis

**Figure 5:**
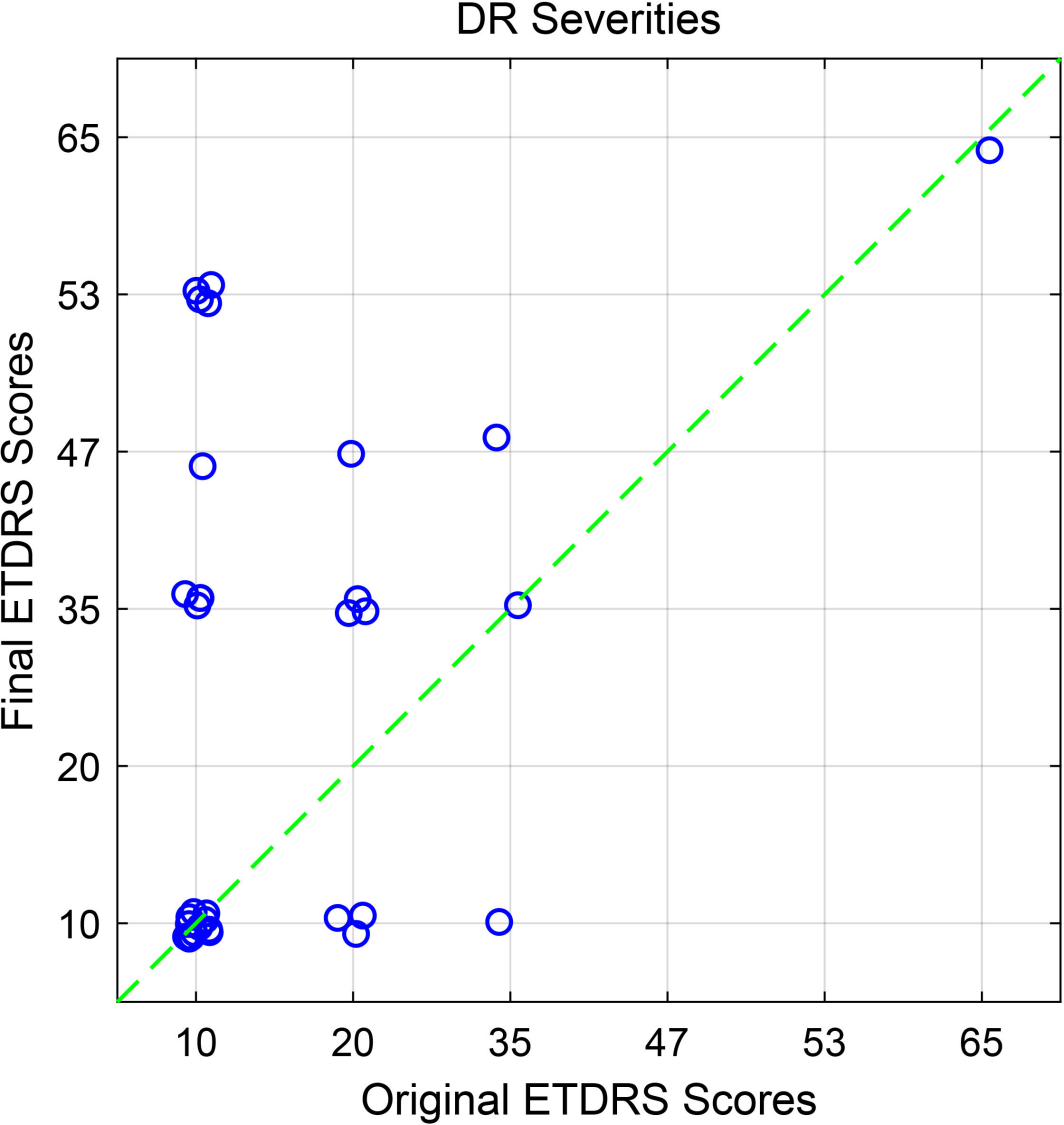
Changes in ETDRS scores between 2013/14 and 2024 for all 32 eyes of the type 2 diabetes patients. The symbol positions have been jittered by a small amount so that they do not completely overlap.

The mean ± SD interval between the first and second visits was 10.47 ± 0.27 years. At the first visit, nine patients showed no signs of DR in either eye, corresponding to an ETDRS severity level of 10 in both eyes. After 10 years, more than half of the participants (10 patients) demonstrated progression of DR by at least one severity level in one eye. **Figure 5** shows the current DR severity levels compared with the baseline. Notably, a few eyes showed mild improvement in DR severity relative to their baseline grading.

**Figure 6:**
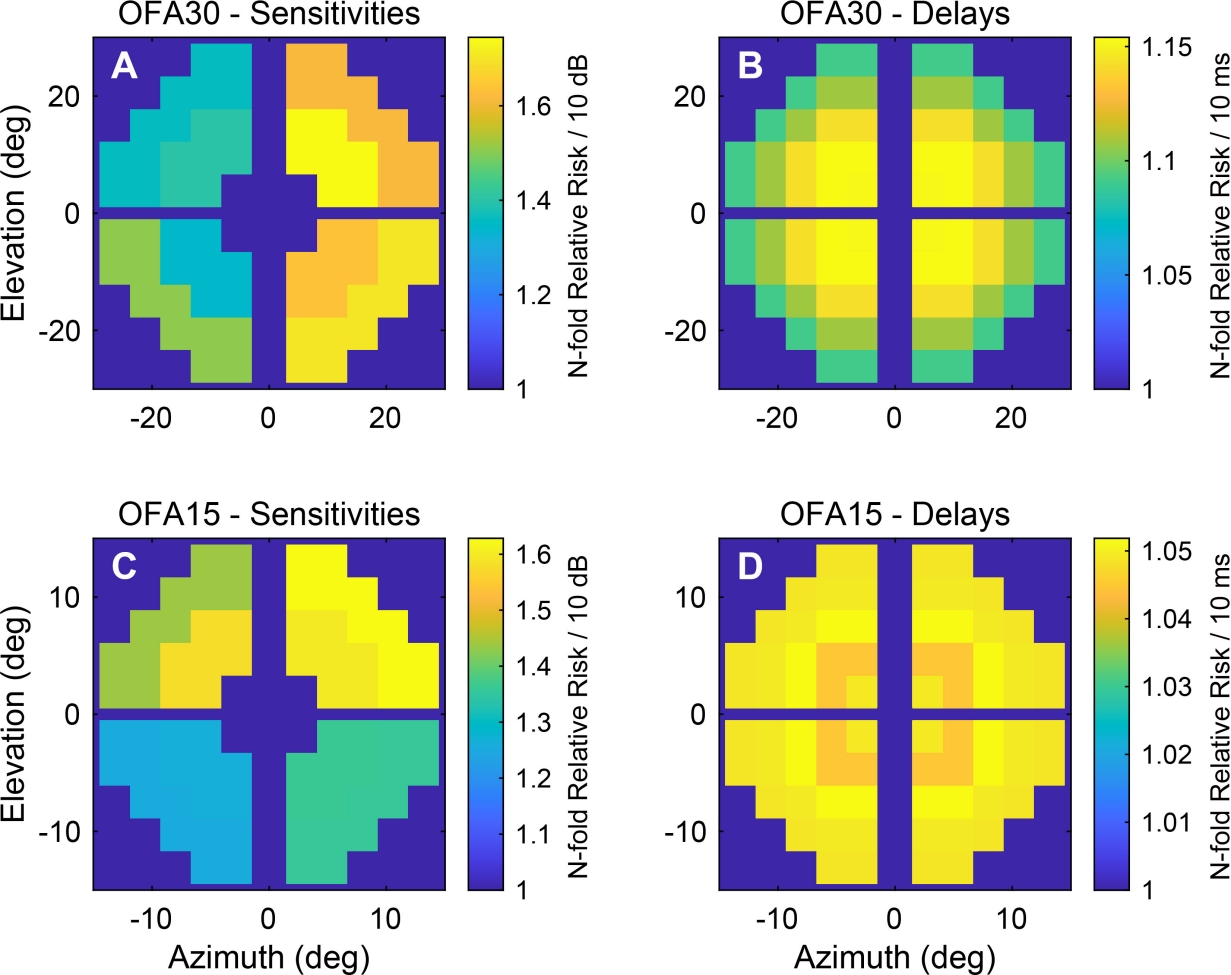
Fitted N-fold relative risks (odds) of diabetic retinopathy progression per 10-dB loss in sensitivity and 10-ms increased delay. **(A)** Relative risk of progression per OFA30 sensitivity loss, **(B)** Relative risk of progression per OFA30 increased delay, **(C)** Relative risk of progression per OFA15 sensitivity loss, **(D)** Relative risk of progression per OFA15 increased delay.

We evaluated the risk of DR progression relative to the per-regional reductions in sensitivity and increases in response delay. In **Figures 6A, C**, each coloured tile represents different visual field regions, with colour intensity indicating the relative increase in risk of DR progression per 10 dB loss in sensitivity at baseline. In the OFA30 sensitivity plot, warmer colours (yellow/orange) in nasal field regions correspond to a higher relative risk (up to 1.74× increase), whereas cooler colours (blue) indicate little or no additional risk. **Figures 6B, D** illustrate the spatial distribution of the added N-fold risk of DR progression associated with a 10-ms increase in response delay at baseline. Regions where delays were more strongly associated with higher progression risk, up to approximately 1.12×, were concentrated in the central regions of OFA30, with the effect gradually decreasing toward the periphery.

### Factors associated with diabetic retinopathy progression

The independent effects of OFA sensitivity and delay TDs, along with demographic and clinical factors in predicting DR progression, were examined using mixed-effects logistic regression models. **Table 2** shows the results based upon the OFA30 sensitivity and delay TDs. To evaluate the effect of visual field eccentricity, the six rings of the 30-2 report (**Figure 2B**) were fitted as categorical factors. In all models, the intercept combines the central ring 1, right eyes of women, and ETDRS severity level 10. The models revealed that older age, being male, higher blood glucose level, ETDRS level 20, and left eyes were significantly associated with lower odds of DR progression, whereas higher estimated glomerular filtration rate (eGFR), diabetes duration, biothesiometry score, and ETDRS level 35 were significantly associated with higher odds of DR progression. In the sensitivity TD models, rings 2 to 6 were significant predictors of DR progression, indicating that increased loss of sensitivity was significantly associated with higher odds of DR progression. Similarly in delay TD models, longer response delays across all rings were significantly associated with higher odds of DR progression. Similar results were obtained when the same models were fitted for the OFA15 TDs, **Supplementary Table S1**.

**Table 2:** Outcomes of mixed-effects logistic regression models showing the significant determinants of DR progression (log odds) using A) OFA30 sensitivity and B) OFA30 delay. For the p-values, 0.000 indicates < 0.0001.

A OFA30 sensitivity				
Variables	Estimate	SE	t-Stat	P-value
(Intercept)	−2.931	0.371	−7.898	0.000
Age	−0.780	0.089	−8.795	0.000
Sex (male)	−2.061	0.237	−8.707	0.000
Eyes (OS)	−1.534	0.109	−14.03	0.000
Blood glucose level	−0.056	0.015	−3.784	0.000
eGFR	0.027	0.003	8.442	0.000
Diabetes duration	0.065	0.015	4.317	0.000
Biothesiometry score	0.123	0.008	15.54	0.000
ETDRS 20	−0.522	0.198	−2.635	0.008
ETDRS 35	1.970	0.225	8.769	0.000
Ring2	0.046	0.020	2.353	0.019
Ring3	0.045	0.012	3.728	0.000
Ring4	0.049	0.011	4.460	0.000
Ring5	0.051	0.010	5.286	0.000
Ring6	0.046	0.008	5.461	0.000
B OFA30 delay				
(Intercept)	−3.565	0.367	−9.711	0.000
Age	−0.783	0.086	−9.085	0.000
Sex (male)	−1.218	0.205	−5.934	0.000
Eyes (OS)	−1.365	0.105	−13.00	0.000
Blood glucose level	−0.049	0.015	−3.234	0.001
eGFR	0.025	0.003	8.057	0.000
Diabetes duration	0.091	0.014	6.561	0.000
Biothesiometry score	0.097	0.008	11.50	0.000
ETDRS 20	−0.750	0.188	−3.985	0.000
ETDRS 35	1.530	0.204	7.504	0.000
Ring 2	0.014	0.004	3.352	0.001
Ring 3	0.014	0.003	5.086	0.000
Ring 4	0.013	0.002	5.585	0.000
Ring 5	0.010	0.002	5.509	0.000
Ring 6	0.009	0.002	5.550	0.000

### Factors associated with changes in sensitivity and delay total deviations

Mixed-effect linear regression models were fitted to identify factors associated with changes in sensitivity and delay TDs between the two visits (**Tables 3A, B**). In the model assessing changes in OFA15 sensitivity (**Table 3A**), older age, higher blood glucose level, longer diabetes duration, and higher body mass index were associated with greater sensitivity loss between visits. In contrast, higher haemoglobin A1c and eGFR were significantly associated with smaller sensitivity changes. **Table 3** also shows that eGFR makes a significant independent contribution to the differences observed between the first and second visit OFA results. For sensitivity, there was a decrease of 0.13 dB per unit increase in eGFR, whereas delays became faster by 0.40 ms per eGFR unit. Furthermore, compared with eyes at ETDRS level 10, those at levels 20 and 35 showed significantly greater losses in sensitivity. Sex and biothesiometry scores were not significant predictors of sensitivity change. It is worth noting that on average PWT2D at ETDRS severity 20 on visit 1 had shorter average delays on visit 2 (35.15 ± 3.25 ms), whereas those that had been at ETDRS 35 had longer delays (7.79 ± 3.80 ms). Similar models examining changes in OFA30 sensitivity and delay TDs between visits are presented in **Supplementary Table S2**.

**Table 3:** Factors associated with between-visit differences in OFA15 sensitivity and delay total deviations.

A Differences in OFA15 sensitivity			
Variables	Estimate	SE	P value
(Intercept)	−0.26	3.08	0.933
Age	0.89	0.30	0.003
Sex (male)	0.84	1.15	0.467
Eye (OS)	0.77	0.33	0.019
Blood glucose level	0.45	0.06	0.000
Haemoglobin A1c 5yr	−1.53	0.19	0.000
eGFR	−0.13	0.01	0.000
Diabetes duration	0.72	0.05	0.000
BMI	0.48	0.04	0.000
Biothesiometry score	0.00	0.02	0.926
ETDRS 20	−5.59	0.69	0.000
ETDRS 35	−11.05	0.77	0.000
B Differences in OFA15 delay			
(Intercept)	−39.02	14.78	0.008
Age	−0.65	1.50	0.665
Sex (male)	4.39	4.78	0.359
Eye (OS)	−7.40	1.59	0.000
Blood glucose level	3.01	0.28	0.000
Haemoglobin A1c 5yr	−7.25	0.93	0.000
eGFR	−0.40	0.06	0.000
Diabetes duration	2.43	0.23	0.000
BMI	2.89	0.21	0.000
Biothesiometry score	0.32	0.11	0.003
ETDRS 20	−35.15	3.25	0.000
ETDRS 35	7.79	3.80	0.041

### Agreement between OFA30 and OFA30-20

**Figure 7:**
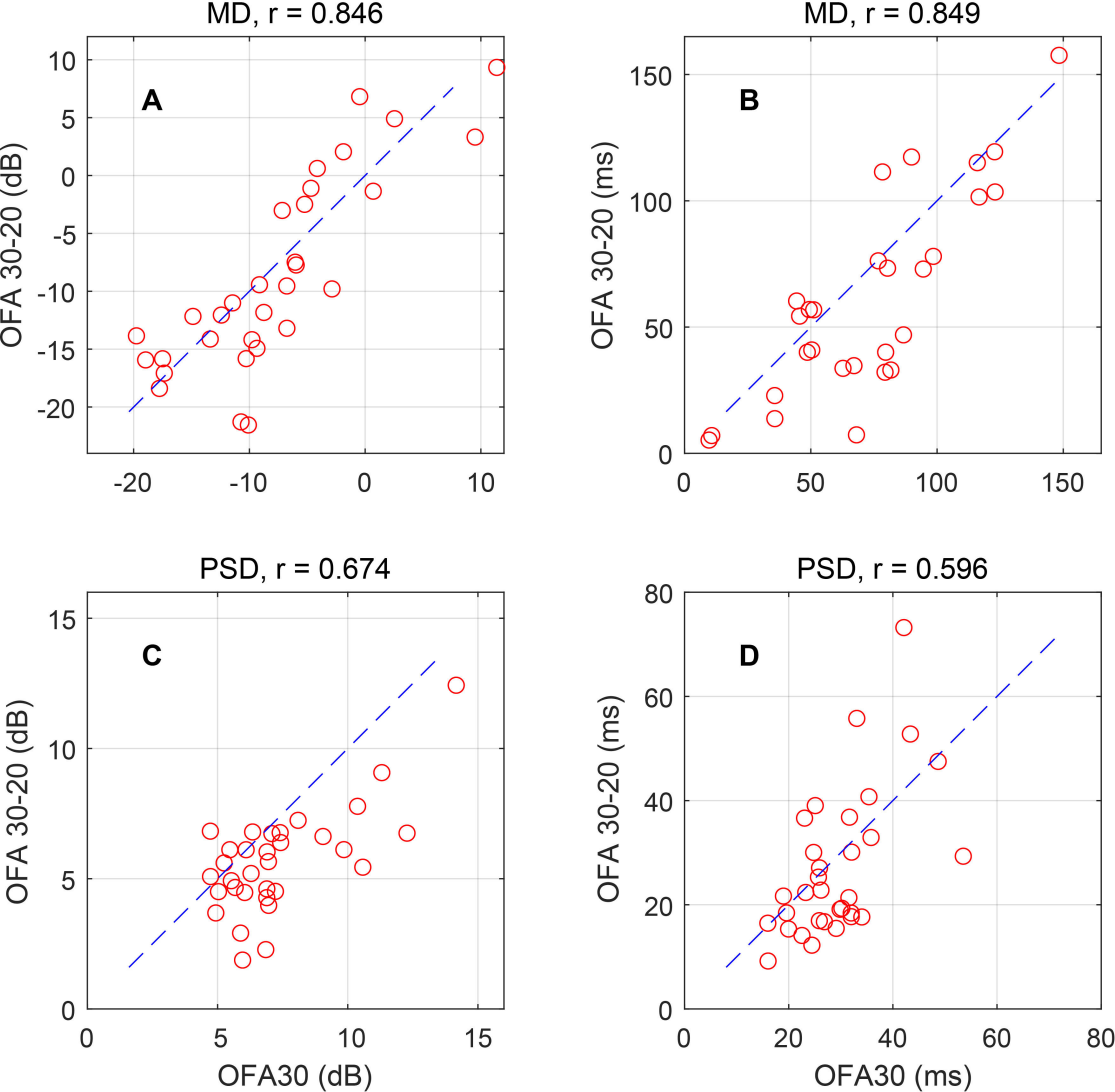
Comparison of OFA30 and OFA 30-20 using mean defects (MDs) **(A, B)** and pattern standard deviations (PSDs) **(C, D)** for sensitivities **(A, C)** and delays **(B, D)**. The measures and their Pearson correlations are shown in the title of each panel. Each panel shows the results for the 32 eyes of visit 2. The blue dashed line represents a slope of 1.

Given that the OFA30 and the OFA30-20 cover the same region of visual space (the central 60 degrees), it is reasonable to ask if there is general agreement between them. The OFA30 field from visit 2 where the fields were compared with the OFA30-20 field was measured on the same day. The summary statistics mean defect (MD) and pattern standard deviation (PSD) were compared (**Figure 7**). The Pearson correlations were all significant at p<0.0003. The MDs were the most similar. Paired t-tests were also done for each comparison. There were no significant differences for MD (dB) or PSD (ms). For MD (ms), the OFA 30 delays were 3.29 ± 4.51 ms longer (p=0.0012, mean ± SE). For PSD (dB), the OFA sensitivities were 5.30 ± 1.65 dB larger. That might be expected given that OFA 30 has a higher spatial resolution and so is able to report on sharper variations in the field.

As for the two macular tests, OFA 10 ETDRS covers only 44.4% of the area of OFA15, so high correlations would not be expected. In fact, the MD (dB) comparison showed a significant correlation, r=0.700 (p<0.0001). The other three possible comparisons showed no significant correlations.

## Discussion

This study assessed retinal function changes over a 10-year period and identified factors predicting DR progression among PWT2D using OFA. At baseline, the cohort included 35 PWT2D with or without DR. Of these, 16 participants were reexamined at the second visit after 10 years. Among those tested at the second visit, more than half (11 patients) had DR in one or both eyes. Moreover, nearly half of examined eyes showed DR progression since the baseline visit (**Figure 5**). DR progression was defined as an increase of one or more levels in ETDRS DR severity compared with the first visit.

We have reported previously that in earlier diabetes, there was an initial stage of hypersensitivity and shorter response delays, which gradually progressed to hyposensitivity and prolonged response delays as the duration and severity of the disease increases (5, 9, 30). Confirming these results, we found a significant association between DR progression and reduced sensitivity and prolonged response delay in all eccentricity rings.

An interesting finding in the OFA literature is that response delays are often more sensitive to early metabolic and retinopathy changes than are sensitivity measures. This is true for multifocal electroretinograms, where delays are somewhat predictive of DR recurrence (31) and onset (32). Recent OFA studies in both type 1 and type 2 diabetes cohorts found that per-region delays provided better discrimination between no-DR and early-DR eyes than amplitude deviations and that delay abnormalities correlated more strongly with metabolic/tissue-injury markers and ETDRS severity scores (7, 33). The current study also revealed that longer central delays were significant predictors of DR progression.

The OFA30 median TD plot revealed progressive sensitivity loss (**Figure 3**), initially in the lower nasal quadrant and later extending to both upper and lower quadrants. This pattern aligns with previous reports of reduced nasal field sensitivity in PWT2D (34). Here, regional sensitivity loss, particularly within the nasal field, was found to be strongly associated with higher risk of DR progression. As shown in **Figure 6A**, reductions in OFA30 sensitivity in the nasal regions corresponded to relative risks of up to 70%. This finding may reflect the non-uniform distribution of vascular lesions in DR. The earliest microvascular changes in DR such as microaneurysms are most commonly located in the temporal retina; this could explain a reduced sensitivity in the nasal fields (34, 35).

The delay TD plot of PWT2D showed defects on the peripheral regions at visit 1 loss (**Figure 3**), with additional central involvement observed at the 10-year follow-up. Our results also suggest that even small increases in response delay around central regions may be clinically significant predictors of DR progression, whereas peripheral delays contribute less to overall risk (**Figure 6B**).

Mixed-effect logistic regressions were fitted to identify independent predictors of DR progression. Older age was associated with reduced odds of DR progression, whereas longer diabetes duration was associated with increasing odds of progression. This was in line with previous studies which reported that the duration of diabetes was more important than age of patients in determining the prevalence and severity of DR (36). Rai et al. similarly reported an increased proportion of patients with hyposensitivity and prolonged delays with increasing duration of diabetes (5). Nitta et al. also found that the duration of type 2 diabetes significantly influences perimetric mean deviation, with visual field defects detected by short-wavelength automated perimetry (SWAP) worsening approximately linearly with disease duration (11). Taken together, this might be because patients who have had long-standing diabetes tend to accumulate more years of exposure to hyperglycaemia and metabolic stress, both well-established risk factors for DR (37). Higher eGFR and biothesiometry score were also associated with increasing odds of DR progression in this study. It is worth noting that OFA tests have been shown to have higher diagnostic power than SWAP or Matrix perimetry (7). OFA testing was also better able to track mild off-axis macular oedema than Matrix perimetry (6).

In our cohort, being male was associated with reduced odds of DR progression. The influence of sex on DR risk has been inconsistently reported in the literature, with some studies finding higher prevalence or faster progression in men, whereas others show greater risk among women (38, 39).

Here, relative to eyes at ETDRS level 10 (no retinopathy), eyes at level 20 showed decreased odds of progression whereas eyes at level 35 showed increased odds. Others have reported that eyes with only isolated microaneurysms (level 20) often remain stable unless microaneurysm turnover (formation/disappearance rates) is high, whereas eyes reaching level 35 (mild non-proliferative DR with additional haemorrhages or exudates) might show a marked increase in risk of worsening (40, 41). How to best gauge the severity of retinopathy is the subject of intense discussion (42, 43).

There was a good agreement between summary measures from the 4th- and 5th-generation tests (**Figure 7**). These more rapid tests sacrifice spatial resolution of the field for speed. In testing both eyes in under 90 s, they are ideal for infirm persons and young people (44, 45).

A major strength of this study is its 10-year prospective follow-up, which offers extensive longitudinal data and enables evaluation of temporal changes in retinal function and disease progression. However, the limitations of this study include the small number of participants returning after 10 years. The study would have been improved by conducting multiple follow-up tests over the 10 years to expand our understanding of functional changes in PWT2D. 10-year structural changes among these participants are not discussed here but will be discussed in a separate article.

In summary, this study assessed visual function changes over the 10-year period among PWT2D using OFA. We observed that the progression of DR was associated with worsening pupillary response amplitudes and delays. In addition, our result indicates that the presence of reduced response amplitude at baseline in the nasal hemifield with OFA30 stimuli was the best predictor of DR progression. This demonstrates the utility and potential of OFA in detecting changes in visual function in PWT2D and monitoring DR progression.

## Data Availability

The raw data supporting the conclusions of this article will be made available by the authors, without undue reservation.
