# Supplementary material for: Objective perimetry and diabetic retinopathy progression: a 10-year follow-up study
Source: Front Endocrinol (Lausanne). 2026 Jan 12;16:1755262. doi: 10.3389/fendo.2025.1755262 (PMC12832394; doi:10.3389/fendo.2025.1755262)
Supplement: Supplementary file 1 [file DataSheet1.pdf]

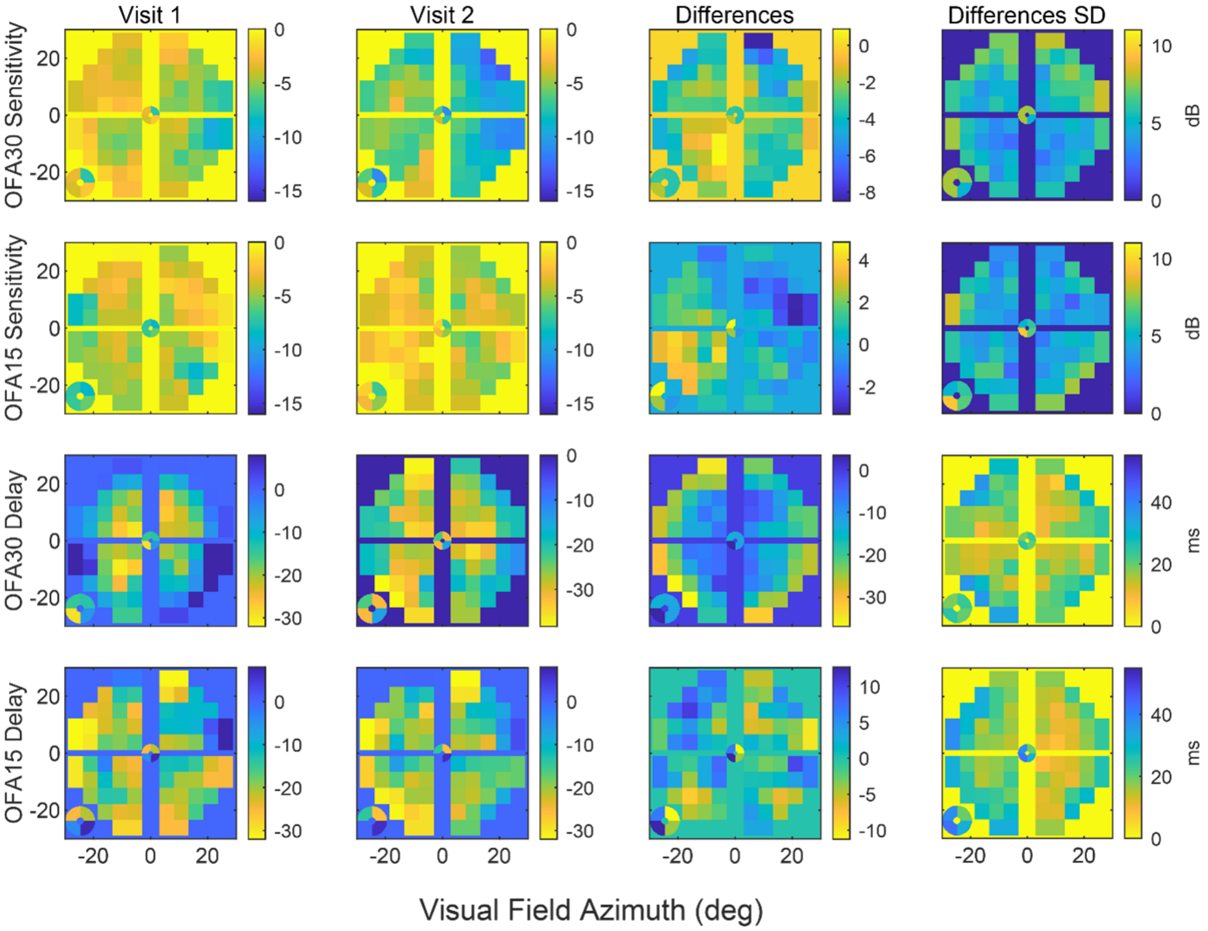

Figure S1: The median sensitivity and delay pattern deviation (PD) data for OFA30 and OFA15 for two visits, their difference and SD. From left to right columns: visit 1 (done in 2013/14), visit 2 (2024), difference and SD of differences. Rows top to down show OFA30 sensitivity, OFA15 sensitivity, OFA30 delay and OFA15 delay data.
