# Supplementary material for: Objective perimetry and diabetic retinopathy progression: a 10-year follow-up study
Source: Front Endocrinol (Lausanne). 2026 Jan 12;16:1755262. doi: 10.3389/fendo.2025.1755262 (PMC12832394; doi:10.3389/fendo.2025.1755262)
Supplement: Supplementary file 2 [file DataSheet2.pdf]

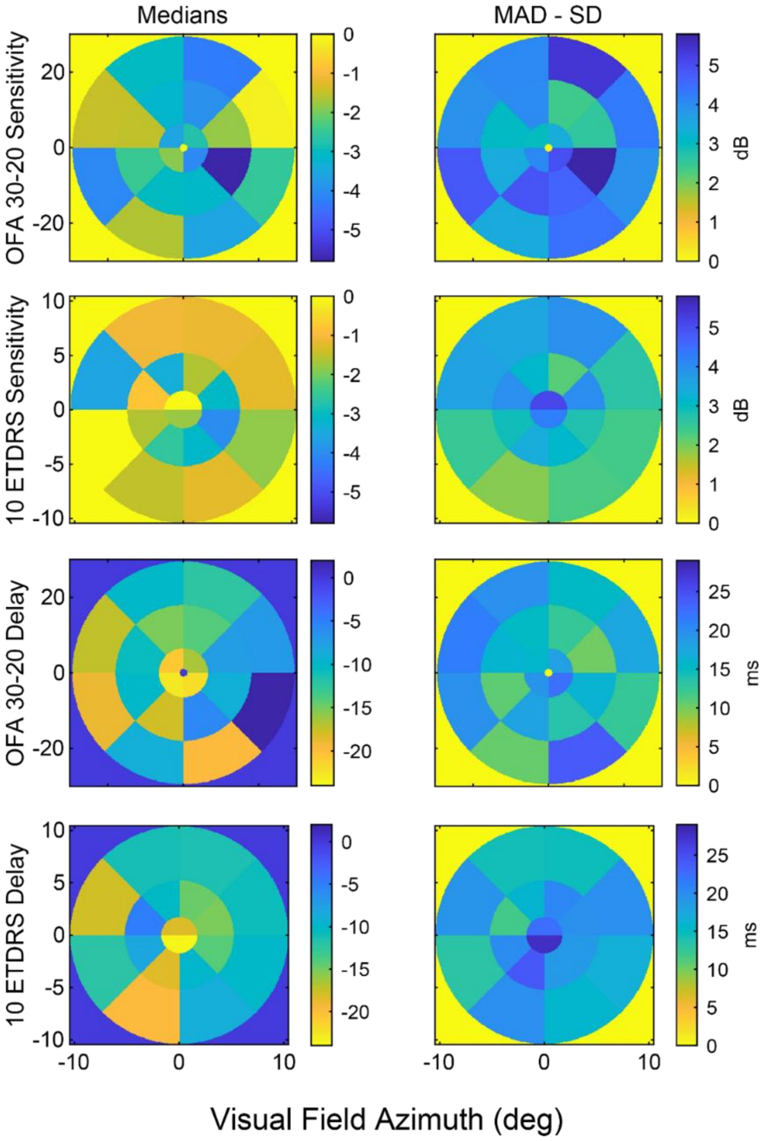

Figure S2: The median sensitivity and delay pattern deviation data for OFA30-20 and OFA10 ETDRS, and their MAD- SD. From left to right columns: medians and MAD-SD. Rows top to down show OFA30-20 sensitivity, OFA10 ETDRS sensitivity, OFA30-20 delay and OFA10 ETDRS delay data.
