# Supplementary material for: Objective perimetry and diabetic retinopathy progression: a 10-year follow-up study
Source: Front Endocrinol (Lausanne). 2026 Jan 12;16:1755262. doi: 10.3389/fendo.2025.1755262 (PMC12832394; doi:10.3389/fendo.2025.1755262)
Supplement: Supplementary file 3 [file DataSheet3.pdf]

Supp Table S1: Outcomes of mixed-effects logistic regression models showing the significant determinants of diabetic retinopathy progression using: A) the OFA15 sensitivity B) the OFA15 delay. For the p-values 0.000 indicates < 0.0001.

**A**	**OFA15 SENSITIVITY**			
**Variables**	**Estimate**	**SE**	**t-Stat**	**p-Value**
(Intercept)	-2.867	0.354	-8.097	0.000
Age	-0.847	0.084	-10.053	0.000
Sex (male)	-2.032	0.169	-12.015	0.000
Eyes (OS)	-1.455	0.107	-13.637	0.000
Blood glucose level	-0.072	0.015	-4.829	0.000
eGFR	0.025	0.003	7.990	0.000
Diabetes Duration	0.085	0.014	5.937	0.000
Biothesiometry score	0.123	0.008	15.870	0.000
ETDRS 20	-0.681	0.187	-3.640	0.000
ETDRS 35	1.771	0.208	8.512	0.000
Ring 2	0.039	0.017	2.294	0.022
Ring 3	0.038	0.010	3.639	0.000
Ring 4	0.039	0.009	4.169	0.000
Ring 5	0.039	0.009	4.536	0.000
Ring 6	0.035	0.008	4.624	0.000
**B**	**OFA15 DELAY**			
(Intercept)	-2.991	0.352	-8.490	0.000
Age	-0.880	0.088	-10.017	0.000
Sex (male)	-1.740	0.192	-9.046	0.000
Eye (OS)	-1.341	0.105	-12.805	0.000
Blood glucose level	-0.049	0.015	-3.307	0.001
eGFR	0.024	0.003	7.592	0.000
Diabetes Duration	0.094	0.014	6.542	0.000
Biothesiometry score	0.111	0.008	13.173	0.000
ETDRS 20	-0.728	0.185	-3.928	0.000
ETDRS 35	1.492	0.202	7.384	0.000
Ring 2	0.005	0.004	1.325	0.185
Ring 3	0.004	0.002	1.957	0.050
Ring 4	0.005	0.002	2.428	0.015
Ring5	0.005	0.002	2.504	0.012
Ring 6	0.005	0.002	2.834	0.005
