# Supplementary material for: Objective perimetry and diabetic retinopathy progression: a 10-year follow-up study
Source: Front Endocrinol (Lausanne). 2026 Jan 12;16:1755262. doi: 10.3389/fendo.2025.1755262 (PMC12832394; doi:10.3389/fendo.2025.1755262)
Supplement: Supplementary file 4 [file DataSheet4.pdf]

**Supp Table S2:** Factors associated with changes in OFA30 sensitivity and delay total deviations.

| **Differences in OFA30 delay** | | | |
|---|---|---|---|
| **Variables** | **Estimate** | **SE** | **P Value** |
| (Intercept) | -48.00 | 12.54 | 0.000 |
| Age | -8.00 | 1.02 | 0.000 |
| Sex (male) | 5.77 | 2.43 | 0.018 |
| Eye (OS) | 0.00 | 1.37 | 0.999 |
| Blood glucose level | -0.02 | 0.24 | 0.948 |
| Haemoglobin A1c 5yr | 2.61 | 0.80 | 0.001 |
| eGFR | -0.09 | 0.05 | 0.111 |
| Diabetes Duration | 0.13 | 0.19 | 0.486 |
| BMI | 1.39 | 0.18 | 0.000 |
| Biothesiometry score | 0.06 | 0.09 | 0.554 |
| ETDRS 20 | -9.76 | 2.67 | 0.000 |
| ETDRS 35 | 8.02 | 3.10 | 0.010 |
| **Differences in OFA30 sensitivity** | | | |
| (Intercept) | -13.72 | 2.98 | 0.000 |
| Age | -1.33 | 0.37 | 0.000 |
| Sex (male) | 0.61 | 0.93 | 0.513 |
| Eye (OS) | 1.56 | 0.32 | 0.000 |
| Blood glucose level | -0.39 | 0.06 | 0.000 |
| Haemoglobin A1c 5yr | 0.25 | 0.19 | 0.178 |
| eGFR | -0.12 | 0.01 | 0.000 |
| Diabetes Duration | 0.72 | 0.04 | 0.000 |
| BMI | 0.49 | 0.04 | 0.000 |
| Biothesiometry score | 0.00 | 0.02 | 0.892 |
| ETDRS 20 | -2.25 | 0.65 | 0.001 |
| ETDRS 35 | -11.97 | 0.76 | 0.000 |
